# Comparison of BCYEα+AB agar and MWY agar for detection and enumeration of *Legionella* spp. in hospital water samples

**DOI:** 10.1186/s12866-021-02109-1

**Published:** 2021-02-16

**Authors:** Savina Ditommaso, Monica Giacomuzzi, Gabriele Memoli, Jacopo Garlasco, Carla M. Zotti

**Affiliations:** Department of Public Health and Pediatrics, University of Turin, 10126 Turin, Italy

**Keywords:** *Legionella*, Culture media, Environmental monitoring

## Abstract

**Background:**

This study illustrates for the first time the performance (sensitivity and selectivity) of the selective medium BCYEα +AB suggested by the new edition of ISO 11731 for legionella isolation and enumeration. We compared the efficacy of the selective BCYEα +AB medium with that of the highly selective MWY medium.

**Results:**

*Legionella* spp*.* was detected in 48.2 and 47.1% of the samples by BCYEα +AB and MWY agar, respectively. For optimal detection of *Legionella* spp., most protocols recommend using selective media to reduce the number of non-*Legionella* bacteria. Agreement between the two media was 86.7%.

**Conclusions:**

According to the results, both media have a very similar performance and they both have advantages and disadvantages over each other. In AB medium there is the risk of being less selective so more interfering microbiota may grow but in MWY medium there is the risk of being too selective. The low selectivity of the AB medium could be resolved if other treatments are applied after filtration, e.g. acid and/or heat treatment, but it must be taken into account that these treatments still reduce the number of viable *Legionella*. In conclusion, we recommend using MWY as a selective medium for the detection of *Legionella* spp. as it is easier discern suspected colonies and facilitate the final *Legionella* spp. count.

**Supplementary Information:**

The online version contains supplementary material available at 10.1186/s12866-021-02109-1.

## Background

*Legionella pneumophila*, which represents the etiological agent responsible for Legionnaires’ disease (LD), was first isolated by McDade et al. in [21]. After its discovery, several occurrences of *Legionella* infection have been reported to be associated with water distribution systems, air conditioning devices, spas and cooling towers [4, 12, 21, 22]. As potable water has long been identified as a potential source of nosocomial and community-acquired LD, environmental surveillance of water systems for the identification of *Legionella* spp. is now being recommended worldwide.

More than 66 *Legionella* species comprising 70 distinct serogroups have been identified to date (https:/l-1psn.dsmz.de/genusl-1egionella). Most *Legionella* species have been isolated from aqueous environments, with at least 30 of them capable of causing infection in humans, mainly in the lower respiratory tract [3].

*Legionella* is a particularly fastidious gram-negative bacterium: in the late 1970s, Feeley and Gorman prepared a new agar medium, cysteine F-G agar-iron, with L-cysteine hydrochloride and soluble ferric pyrophosphate [11], while Feeley et al. modified the medium by replacing casein acid hydrolysate with yeast extract and by adding activated charcoal as a scavenger of radicals and peroxides [10] and the resulting charcoal yeast extract agar (CYE) medium enhanced the growth of *Legionella* spp. Later on, Pasculle et al. supplemented the CYE medium with N-(2-Acetamido)-2-aminoethanesulfonic acid (ACES) buffer, thereby obtaining buffered charcoal yeast extract agar (BCYE) which, under aerobic conditions, enabled a better recovery of *Legionella* [27], and Edelstein further increased this medium’s sensitivity by adding α-ketoglutarate (i.e., BCYEα agar) [8].

Since then, numerous selective media based on different inhibiting agents have been developed so as to limit the development of interfering microbiota that may reduce or inhibit the recovery of these bacteria. Notable among these are GVPC agar, a BCYEα medium supplemented with 3 g/l glycine, 0.001 g/l vancomycin, 80,000 IU/l polymyxin B and 0.08 g/l cycloheximide, and the modified *Wadowsky*–*Yee (*MWY*)* medium [9, 32], another BCYEα medium containing, differently from GVPC medium, 3 g/l glycine, 50,000 IU/l polymyxin B, 0.001 g/l vancomycin, 0.08 g/l anisomycin and the colors bromothymol blue and bromocresol purple, which stain the colonies and aid in the identification of the organisms. For optimal detection of *Legionella* spp. via reduction of non-*Legionella* bacteria, most protocols, i.e. Health Protection Agency [15], AFNOR [1], ISO [16] and Centers for Disease Control and Prevention [2], recommend using selective agar consisting of buffered charcoal yeast extract (BCYEα) agar containing 1 g/l alpha-ketoglutarate supplemented with glycine, vancomycin, polymyxin B and cycloheximide (GVPC). Although this medium is now widely used in laboratories worldwide, the presence of contaminating bacterial flora in water samples frequently reduces the recovery of *Legionella* spp. due to overgrowth or inhibition [29].

Since 1997, our laboratory has been conducting *Legionella* spp. testing on environmental samples using two types of medium: BCYEα and MWY [5]. We adopted MWY because its dyes stain the colonies with better differentiation [9, 30]. The combined use of BCYEα agar with selective agar for improved *Legionella* detection has been recently (2017) recommended by the second edition of ISO 11731 [17]. This new edition also proposes three different types of selective media for *Legionella* isolation: GVPC or MWY agar for water samples with a high concentration of interfering microbiota, and BCYEα +AB agar (i.e., natamycin, cefazolin and polymyxin B) for samples containing low concentrations of interfering microbiota. This latter selective medium was first recommended by the Dutch Standard NEN 6265 [26].

The goal of this study was to compare the performance of the BCYEα +AB vs. MWY agar in terms of sensitivity and selectivity in suppressing the growth of interfering microbiota from water samples taken from hospital water supplies.

## Results

A total of 263 hot water samples, all taken from hospital potable water faucets, were cultured on two different media (i.e., BCYEα +AB and MWY) to isolate *Legionella* spp. Of these, 143 (54.4%) were *Legionella* positive at least on one medium. The distribution of the results between the two media is shown in Table 1.

**Table 1:** Recovery of *Legionella* spp. by two different detection media

MWY agar	BCYEα + AB agar	Total	(%)
*negative*	*negative*	111	42.2
*negative*	*positive*	19	7.2
*positive*	*positive*	108	41.1
*positive*	*negative*	13	4.9
*positive*	*overgrowth*^a^	3	1.1
*negative*	*overgrowth*^a^	4	1.5
*overgrowth*^a^	*overgrowth*^a^	5	1.9
		**263**	

^a^overgrowth of background flora. Corresponding samples were considered as Legionella-negative as described in the Results

A small minority of the specimens (5/263) contained Gram-negative bacteria that were not inhibited by either selective medium (i.e., MWY and BCYEα +AB), while 7 further samples contained flora whose growth was inhibited by the MWY selective medium but not by BCYEα+AB (Table 1). Therefore, the evaluation of the presence of *Legionella* in our samples (and enumeration of *Legionella* spp. colonies for positive samples) was possible for 98.1% (258/263) of the samples seeded on MWY agar and 95.4% (251/263) of those seeded on BCYEα +AB agar, whereas all samples contaminated by overgrowth of Gram-negative bacteria were deemed as *Legionella* negative.

Agreement between the two methods was 86.7%. Calculation of Cohen’s κ-coefficient showed good concordance (κ = 0.733) (Table 2).

**Table 2:** Comparison of *Legionella* spp. recovery obtained with different culture media

		BCYEα + AB agar		
		***Positive (n)***	***Negative (n)***	***Total (n)***
**MWY agar**	***Positive (n)***	108	16	**124**
	***Negative (n)***	19	120	139
	***Total (n)***	**127**	136	263

Agreement = 86.7%; κ = 0.733

Table 3 compares the results concerning Legionella counts of the 108 concordant positive samples.

**Table 3:** Comparison between *Legionella* counts (CFU/l) of concordant positive samples

	No. (%) of samples	MWY agar			BCYEα + AB agar		
		Geometric mean	Median	Range	Geometric mean	Median	Range
**Higher counts on MWY**	37 (34.3%)	1.7 × 10^3^	1.6 × 10^3^	2.0 × 10^2^ - 1.7 × 10^4^	1.0 × 10^3^	9.0 × 10^2^	5.0 × 10^1^ - 1.6 × 10^4^
**Higher counts on BCYEα + AB**	56 (51.8%)	8.1 × 10^2^	9.5 × 10^2^	5.0 × 10^1^ - 8.3 × 10^3^	1.3 × 10^3^	1.2 × 10^3^	1.0 × 10^2^ - 1.7 × 10^4^
**Counts on MWY agar = counts on on BCYEα AB**	15 (13.9%)	6.1 × 10^2^	3.0 × 10^2^	5.0 × 10^1^ - 3.3 × 10^5^	6.1 × 10^2^	3.0 × 10^2^	5.0 × 10^1^ - 3.3 × 10^5^

The most frequently isolated species was *L. pneumophila* (59.4%) and was detected equally using the two agar media. In 29 of the 143 positive water samples, *L. pneumophila* grew with associated non-*pneumophila Legionella* species*.* The most frequently isolated serogroups were *L. pneumophila* serogroup 6 and serogroup 1. The distribution of the results is shown in Table 4.

**Table 4:** Frequency of *L. pneumophila* and non-*pneumophila Legionella* species detection from hospital water samples

Species	Serogroups	Total (n)
	*L. pneumophila* sg 1	22
	*L. pneumophila* sg 2	4
*L. pneumophila*	*L. pneumophila* sg 3	5
	*L. pneumophila* sg 6	40
	*L. pneumophila* sg 7–14	14
*L.* species non-*pneumophila*	autofluorescens	35
	Other	23
		**143**

**Fig. 1:**
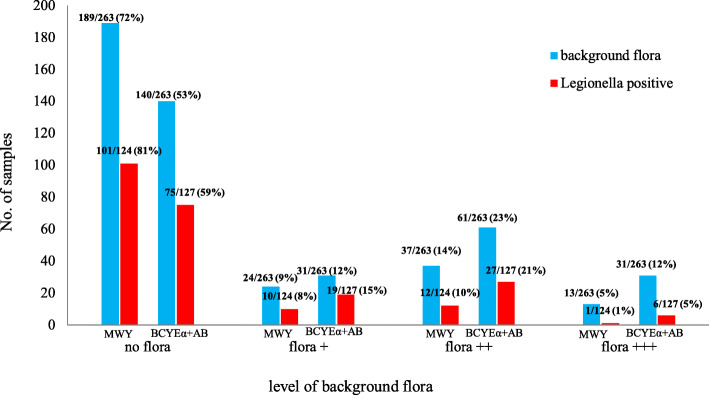
Comparison of selective ability of MWY agar and BCYEα+AB. The presence of *Legionella* spp. and relationship with other bacteria. Background flora was measured through semi-quantitative counting, where zero was no background flora and 3+ was massive contamination (see Additional file 1)

Among the 124 samples who tested *Legionella* spp. positive on MWY agar, 23 showed the presence of accompanying microbiota (18.5%), whereas accompanying microbiota was observed in 52 out of 127 *Legionella* spp. positive samples (40.9%) on BCYEα+AB agar (Fig. 1).

**Fig. 2:**
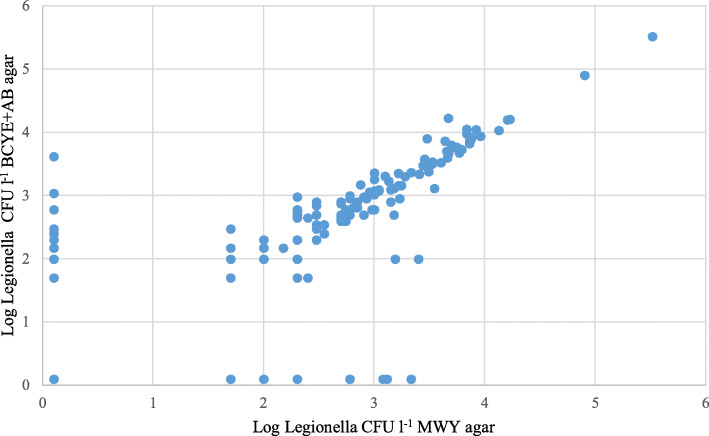
Comparison of *Legionella* counts (CFU/l) on different media**.** Scatter plot in logarithmic scale comparing *Legionella* spp*.* detection by MWY and BCYEα +AB counts. Kendall’s tau correlation coefficient was computed taking into account only samples positive for *Legionella* spp. on both media

To determine whether the medium composition had an effect on *Legionella* spp. enumeration, we compared the final counts obtained from each medium through the Wilcoxon signed-rank test, which showed absence of significant correlation between the counts and the medium (*p* = 0.09934). The same analysis yielded a statistically significant difference in non-*Legionella* flora growth between the MWY and BCYEα +AB media (*p* < 0.0001). Furthermore, the counts from the 108 positive samples on both agars were compared by evaluating Kendall’s *tau* correlation coefficient, which showed a high degree of correlation (*τ* = 0.7852, *p* < 0.0001) (Fig. 2).

## Discussion

*Legionella* spp. is characterized by an extended lag period of growth, requiring at least 3 days to produce visible colonies on BCYEα agar, so its detection is often hindered by the presence of other bacteria. In this regard, the selective media MWY and GVPC, recommended when isolating these bacteria from environmental specimens, rarely achieve a good balance between false positive and false negative results, which reduces the recovery of the target organism (i.e., *Legionella* spp.) [13, 19]. In a previous study, we demonstrated that these limitations can be overcome by using BCYEα agar, which can significantly improve isolation and enumeration of *Legionella* spp. [5]**.**

More recently, the new edition of ISO 11731 [17] has proposed the use of three different types of selective media for detecting *Legionella* species: GVPC or MWY agar for water samples with a high concentration of background flora and BCYEα+AB agar for samples with a low concentration of interfering microorganisms. However, given that the selective culture of *Legionella* may also be influenced by the degree of susceptibility of the microorganisms to antimicrobials, which varies according to their physiological state, here we inquired whether the use of BCYEα +AB agar could improve the sensitivity of *Legionella* spp. detection in environmental water samples over that of current methods. To our knowledge, this is the first study addressing detection and enumeration of *Legionella* species on BCYEα +AB medium.

Agreement between the results obtained with the two media was 86.7%. Importantly, parallel seeding showed that the number of *Legionella* CFUs on BCYEα +AB agar was not significantly higher than the number of CFUs found on MWY agar (medium with higher concentrations of antibiotics and antifungals) (*p* = 0.09934), indicating that there are no significant differences in sensitivities between the two selective procedures. Thus, our results demonstrate that both selective media are suitable for primary plating of environmental specimens for the isolation of *Legionella* spp.

According to the results, both methods have a very similar performance and they both have advantages and disadvantages over each other. In AB media there is the risk of being less selective so more interfering microbiota may grow but in MWY media there is the risk of being too selective and *Legionella* spp. cells that are harmed or not that much active in the water systems would not grow. The low selectivity of the AB medium could be resolved if other treatments are applied after filtration, e.g. acid and / or heat treatment, but it must be taken into account that these treatments still reduce the number of viable *Legionella*.

## Conclusions

In conclusion, we recommend using MWY as a selective medium for the detection of *Legionella* spp. as it is easier discern suspected colonies and facilitate the final *Legionella* spp. count (see Additional file 2).

## Methods

### Environmental sampling

Hot water samples were collected from in-building distribution systems of healthcare facilities of acute care hospitals while conducting environmental monitoring programs for Legionella spp. detection. All samples were collected from water faucets without previously running the water and without flaming the outlet point, in accordance with the Italian Guidelines for water sampling in common use conditions, namely ‘instantaneous sampling’, to simulate theoretical user exposure [23]. Each sample was collected in sterile one-liter plastic bottles. Sodium thiosulphate solution (100 mg/l) was added to the samples to neutralize free chlorine in treated water supplies. The samples were then transported to the laboratory at room temperature and processed on the day of collection.

### Laboratory procedure

Analyses for the quantification of Legionella spp. were performed according to an internal method [5]. Water samples were cultured onto MWY agar (Xebios Diagnostics GmbH, Düsseldorf, Germany), i.e., BCYEα agar with the addition of glycine (3 g/l), polymyxin B (50,000 IU/l), vancomycin (0.001 g/l), anisomycin (0.08 g/l), bromothymol blue (0.01 g/l) and bromocresol purple (0.01 g/l, colors which help distinguish more easily between *Legionella* and non-*Legionella* bacteria), and onto BCYEα+AB (Xebios Diagnostics GmbH, Düsseldorf, Germany), consisting of natamycin (65 mg/l), cefazolin (9 mg/l) and polymyxin B (80,000 IU/l).

Briefly [5], the one liter water samples was concentrated 100-times by filtration using 0.22 μm polycarbonate filter (Millipore, Billerica, MA, USA). After filtration, the membrane filter was aseptically placed in one of the bottom corners of a stomacher bag with 10 ml Page solution (pH 6.8) and rubbed for 1 min, in order to detach bacteria. A 0.2 ml volume of the concentrated sample was placed in duplicate on plates of MWY and BCYEα+AB agar, and the plates were then incubated at 36 °C with 2.5% CO_2_ for 10 days. The plates were checked at days 2, 3, 5 and then at the end of the incubation period. Presumptive *Legionella* colonies were confirmed by subculturing on blood agar (Oxoid Ltd., Basingstoke, UK) and BCYEα agar. Colonies grown on MWY agar or BCYEα+AB agar were identified according to ISO 11731 procedure (one colony type: three presumptive colonies were subcultured; more colonies types: at least one colony from each type were subcultured) by means of an agglutination test (Legionella latex test; Oxoid). The latex test allows separate identification of *L. pneumophila* serogroup 1 and serogroups 2 to 14 and detection of seven other non-*pneumophila Legionella* spp. (*L. longbeachae* 1 and 2, *L. bozemanii* 1 and 2, *L. dumoffii*, *L. gormanii*, *L. jordanis*, *L. micdadei*, and *L. anisa*) [7, 14, 18, 20, 25, 31]. Colonies recognized as L. pneumophila serogroup 2 to 14 were further tested with single Legionella agglutination latex reagents (Pro-Lab Diagnostics, Richmond Hill, Canada) for the identification of the different L. pneumophila serogroups. If the agglutination test with the colonies was negative, the isolates underwent a polymerase chain reaction test (in-house PCR) for the amplification of the 16S rRNA gene of *Legionella* spp., as previously described [24]. The plate with the higher number of confirmed colonies was used to estimate the number of *Legionella* spp. in the original sample. *Legionella* spp. concentrations in water samples are expressed in colony forming unit per liter (CFU/l). According to the concentration procedure, the detection limit of our method is 50 CFU/l. The presence of background flora was measured through semi-quantitative counting [6]: four categories were determined according to visual density of colonies spread onto the plate, where zero was no background flora and 3+ was massive contamination (See Additional file 1).

### Statistical analysis

Statistical analyses were performed using the statistical software R (“stats” package, version 3.6.2) [28]. Agreement between the two media was assessed by comparing the results of the MWY and BCYEα+AB media on two-by-two contingency tables, through Cohen’s κ coefficient. The non-parametric Wilcoxon signed-rank test was applied to all samples in order to compare differences in microbial loads between MWY agar and BCYEα+AB agar for both Legionella spp. and background flora. Kendall’s *tau* correlation coefficient was employed to compare the ability of the two media to cultivate *Legionella*. The analysis was performed by taking into account only samples positive for *Legionella* spp. on both media, after checking the preliminary assumptions.

## Supplementary Information

**Additional file 1.** Background flora. Examples of plates with different level of background flora from complete absence (zero) to massive contamination (3+).

**Additional file 2.** Paired photos of MWY BCYEα+AB obtained during incubation period of inoculated plates. Examples of Concordant Positive Samples (MWY agar on the left and BCYEα+AB agar on the right) (pagg1,2). Samples Positive Only on MWY agar (pag 3). Samples Positive Only on BCYEα+AB agar (pag 4). Examples of Samples With Overgrowth only on Bcye+Ab Agar; Examples of Samples With Overgrowth on Both Agar Media (pag 5).

## Data Availability

The dataset used and/or analysed during the current study are available from the corresponding author on reasonable request.

## Abbreviations

BCYE: Buffered Charcoal Yeast Extract agar
BCYEα: Buffered Charcoal Yeast Extract agar with α-ketoglutarate;
BCYEα+AB: Buffered Charcoal Yeast Extract agar with α-ketoglutarate and Antibacterials
CYE: Charcoal Yeast Extract agar
GVPC: Glycine Vancomycin Polymyxin and Cycloheximide agar
LD: Legionnaires’ Disease
MWY: Modified Wadowsky-Yee medium
PCR: Polymerase Chain Reaction
rRNA: Ribosomal RNA
