# Supplementary material for: Comparison of BCYEα+AB agar and MWY agar for detection and enumeration of Legionella spp. in hospital water samples
Source: BMC Microbiol. 2021 Feb 16;21:48. doi: 10.1186/s12866-021-02109-1 (PMC7885575; doi:10.1186/s12866-021-02109-1)
Supplement: Supplementary file 1 — Additional file 1. Background flora. Examples of plates with different level of background flora from complete absence (zero) to massive contamination (3+). [file 12866_2021_2109_MOESM1_ESM.pdf]

Examples of plates with different level of background flora from complete **absence** (zero) to massive contamination (3+)

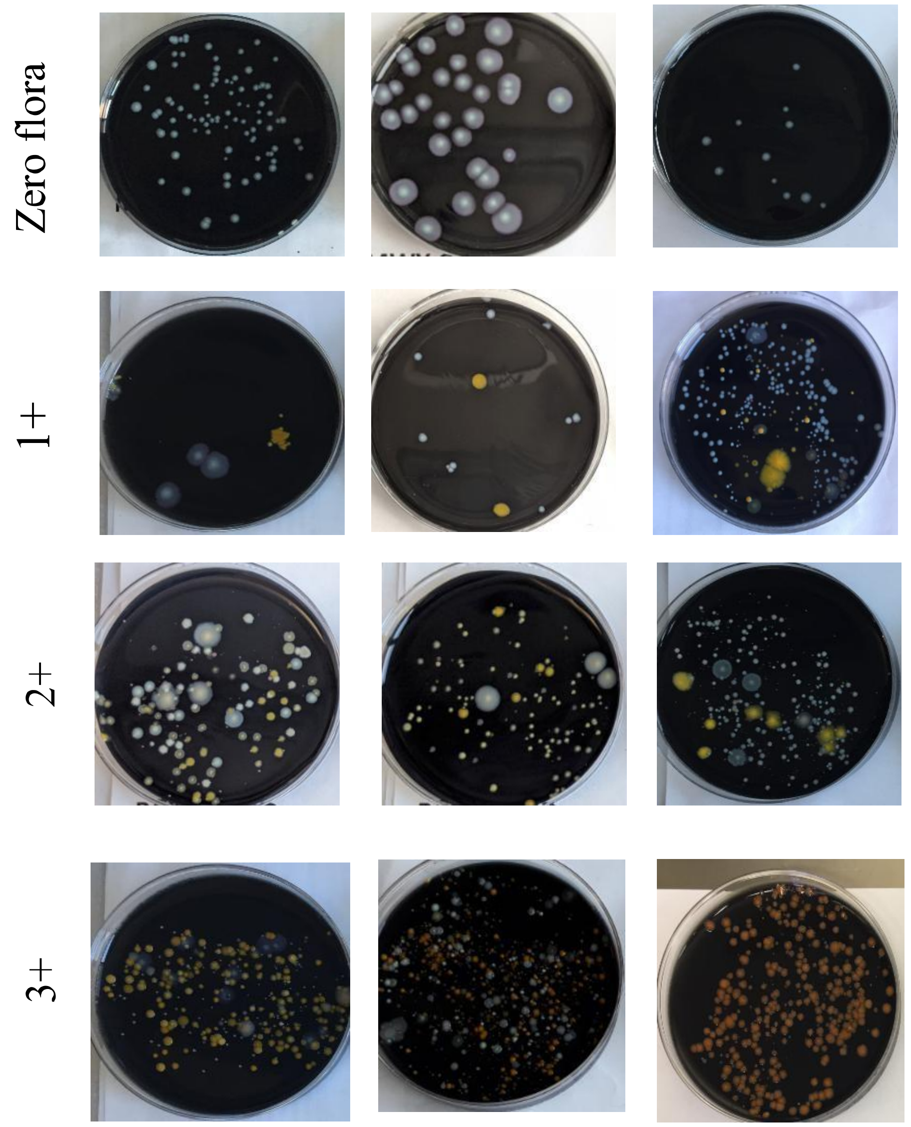

Comparison of BCYEŬ+AB Agar and MWY agar for Detection and Enumeration of Legionella spp. in hospital water samples

Savina Ditommaso, Monica Giacomuzzi, Gabriele Memoli, Jacopo Garlasco and Carla M. Zotti1

Corresponding author: savina.ditommaso@unito.it; Department of Public Health and Pediatrics, University of Turin, 10100 Turin, Italy
