# Supplementary material for: Comparison of BCYEα+AB agar and MWY agar for detection and enumeration of Legionella spp. in hospital water samples
Source: BMC Microbiol. 2021 Feb 16;21:48. doi: 10.1186/s12866-021-02109-1 (PMC7885575; doi:10.1186/s12866-021-02109-1)
Supplement: Supplementary file 2 — Additional file 2. Paired photos of MWY BCYEα+AB obtained during incubation period of inoculated plates. Examples of Concordant Positive Samples (MWY agar on the left and BCYEα+AB agar on the right) (pagg1,2). Samples Positive Only on MWY agar (pag 3). Samples Positive Only on BCYEα+AB agar (pag 4). Examples of Samples With Overgrowth only on Bcye+Ab Agar; Examples of Samples With Overgrowth on Both Agar Media (pag 5). [file 12866_2021_2109_MOESM2_ESM.pdf]

EXAMPLES of CONCORDANT POSITIVE SAMPLES (MWY agar on the left and BCYE+AB agar on the right)

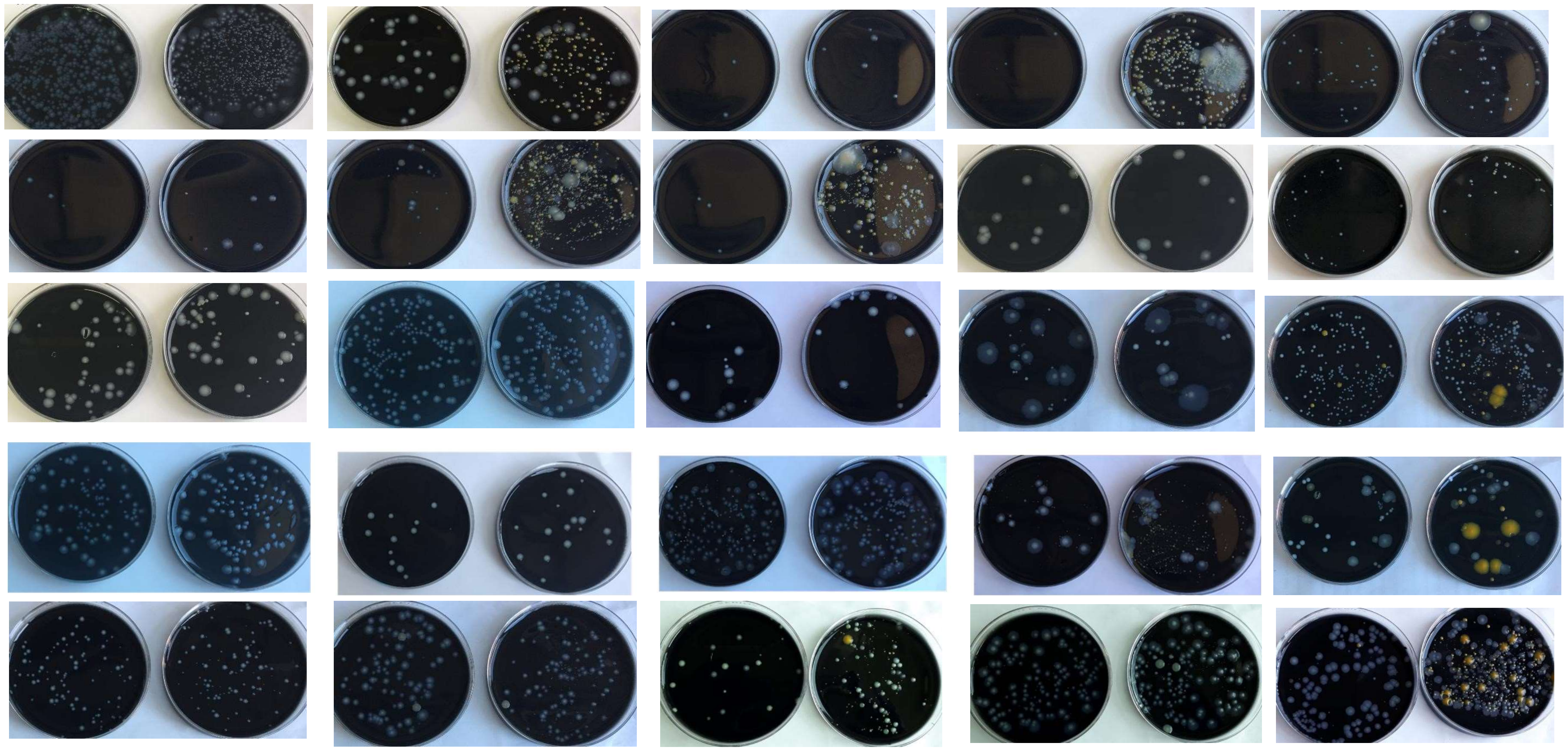

EXAMPLES of CONCORDANT POSITIVE SAMPLES (MWY agar on the left and BCYE+AB agar on the right)

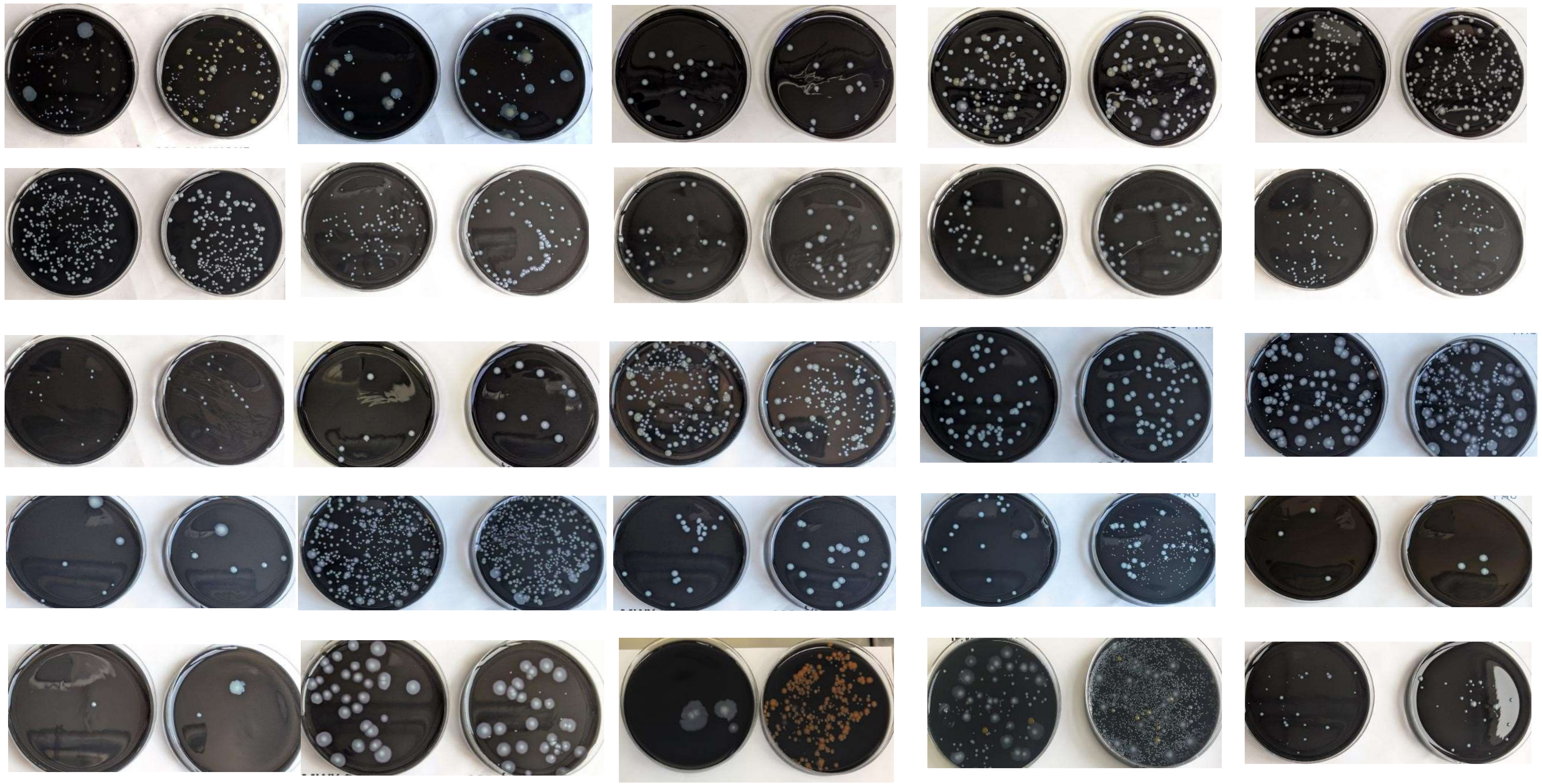

SAMPLES POSITIVE ONLY ON MWY agar

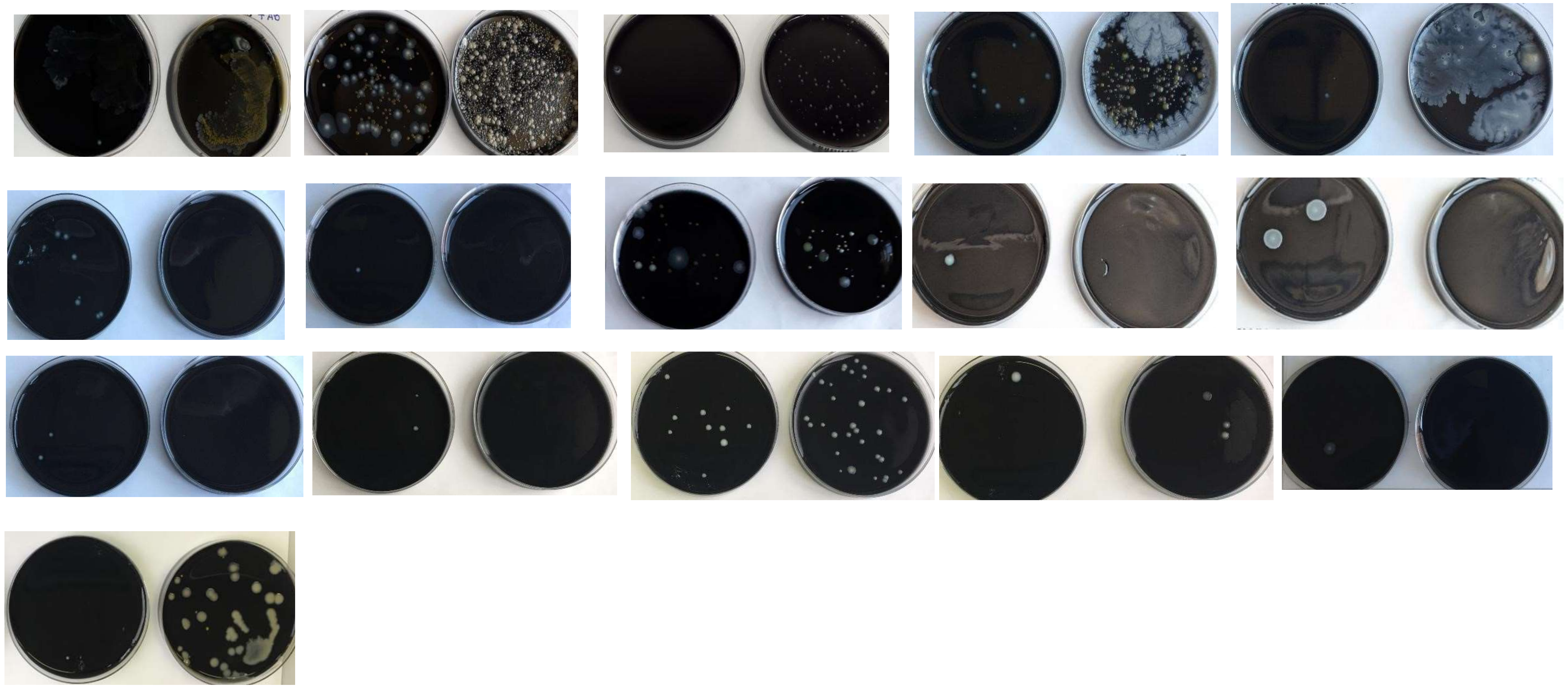

SAMPLES POSITIVE ONLY ON BCYE+AB agar

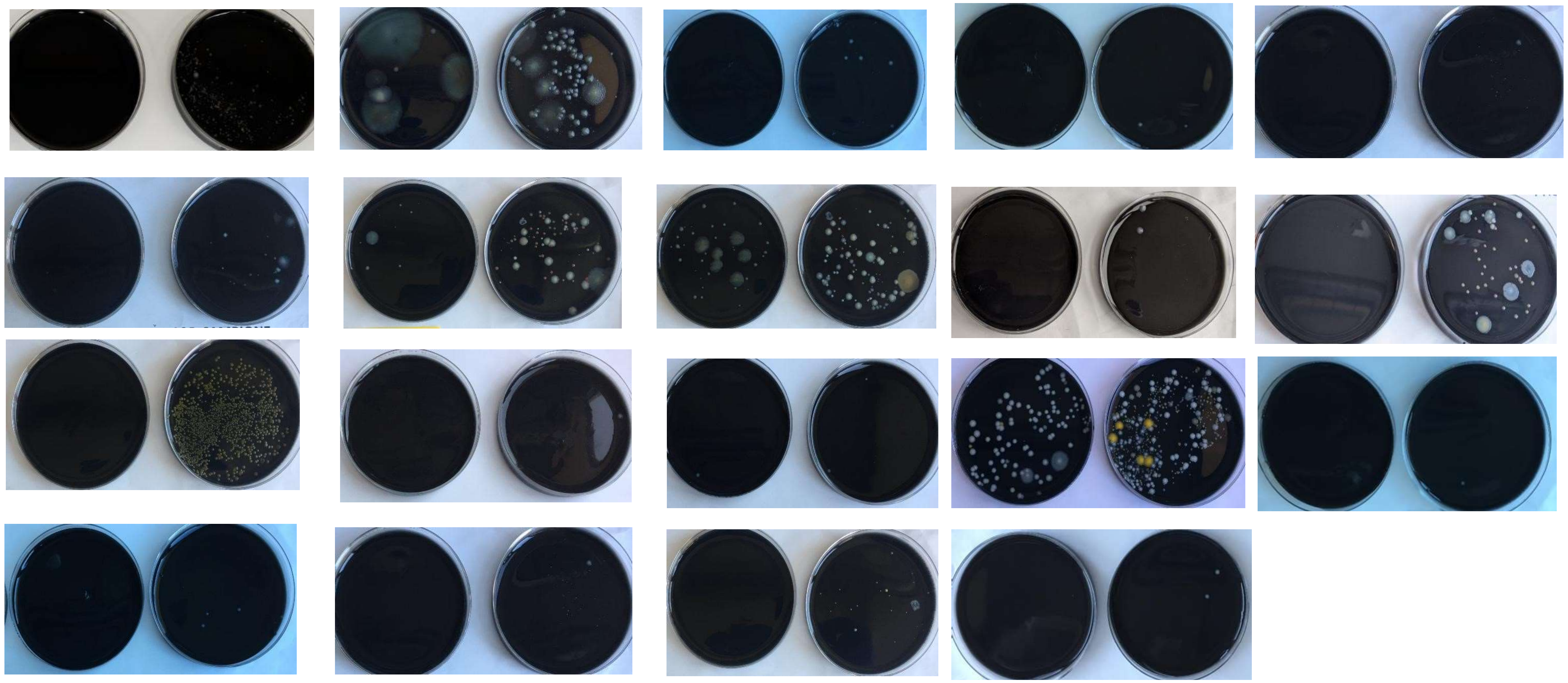

EXAMPLES OF SAMPLES WITH OVERGROWTH ONLY ON BCYE+AB agar

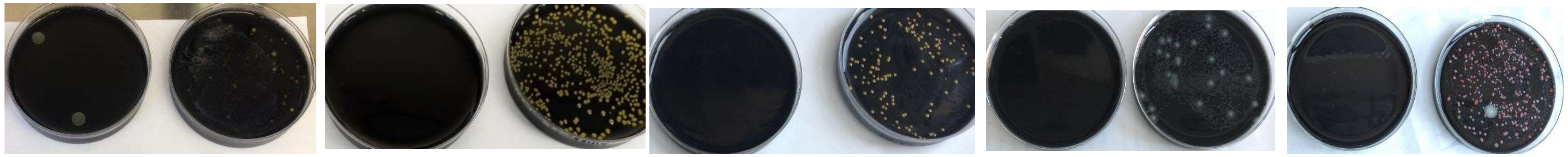

EXAMPLES OF SAMPLES WITH OVERGROWTH ON BOTH AGAR MEDIA

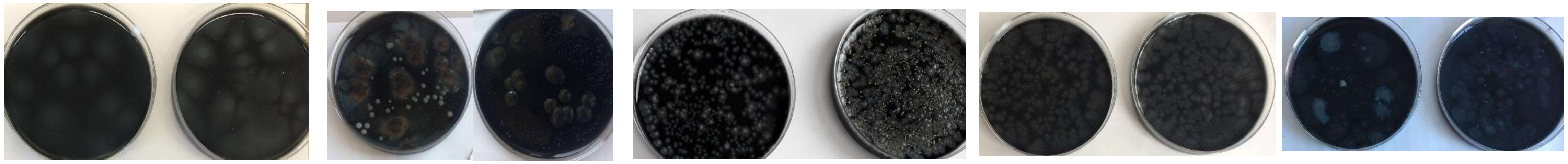

Comparison of BCYEα+AB Agar and MWY agar for Detection and Enumeration of Legionella spp. in hospital water samples

Savina Ditommaso, Monica Giacomuzzi, Gabriele Memoli, Jacopo Garlasco and Carla M. Zotti1

Corresponding author: savina.ditommaso@unito.it; Department of Public Health and Pediatrics, University of Turin, 10100 Turin, Italy
